# Retraction: FOXO4 overexpression suppresses hypoxia-induced-MCF-7 cell survival and promotes apoptosis through the HIF-2α/Bnip3 signal pathway

**DOI:** 10.1039/d1ra90014e

**Published:** 2021-01-22

**Authors:** Laura Fisher

**Affiliations:** Royal Society of Chemistry Thomas Graham House, Science Park, Milton Road Cambridge CB4 0WF UK advances-rsc@rsc.org

## Abstract

Retraction of ‘FOXO4 overexpression suppresses hypoxia induced-MCF-7 cell survival and promotes apoptosis through HIF-2α/Bnip3 signal pathway’ by Yan Qiao *et al.*, *RSC Adv.*, 2019, **9**, 25912–25918, DOI: 10.1039/C9RA04380B.

The Royal Society of Chemistry hereby wholly retracts this *RSC Advances* article due to concerns with the reliability of the data. The images in the article, and the raw data provided by the authors, were screened by an image integrity expert who identified many instances of image manipulation affecting the western blots.

The backgrounds to the western blots in Fig. 1 show splice marks between each pair of bands. The top edge of the FOXO4 control band appears manipulated.

In Fig. 2, the top and bottom edges of the two middle bands (Hypoxia + pcDNA3.1) are identical. Analysis of the bands indicates that they are likely to be the stretched versions of the same band.

Repeating sections of background have been identified in the HIF-2α and Bnip3 panels in Fig. 4. Repeating sections can also be observed in the backgrounds of both blots in Fig. 5.

Given the significance of the concerns about the validity of the data in the article, the findings presented in this paper are not reliable.

The authors have been informed but have not responded to any correspondence regarding the retraction.

Signed: Laura Fisher, Executive Editor, *RSC Advances*

Date: 7^th^ January 2021

## Supplementary Material

